# Quality of Life, Postoperative Pain, and Lymph Node Dissection in a Robotic Approach Compared to VATS and OPEN for Early Stage Lung Cancer

**DOI:** 10.3390/jcm10081687

**Published:** 2021-04-14

**Authors:** Pierluigi Novellis, Patrick Maisonneuve, Elisa Dieci, Emanuele Voulaz, Edoardo Bottoni, Sara Di Stefano, Michela Solinas, Alberto Testori, Umberto Cariboni, Marco Alloisio, Giulia Veronesi

**Affiliations:** 1 Division of Thoracic Surgery, IRCCS San Raffaele Scientific Institute, 20132 Milan, Italy; dieci.elisa@hsr.it (E.D.); veronesi.giulia@hsr.it (G.V.); 2 Division of Epidemiology and Biostatistics, IEO, European Institute of Oncology IRCCS, 20141 Milan, Italy; patrick.maisonneuve@ieo.it; 3 Division of Thoracic and General Surgery, Humanitas Clinical and Research Center, IRCCS, Rozzano, 20089 Milan, Italy; emanuele.voulaz@humanitas.it (E.V.); edoardo.bottoni@humanitas.it (E.B.); sara.di_stefano@humanitas.it (S.D.S.); alberto.testori@humanitas.it (A.T.); umberto.cariboni@humanitas.it (U.C.); marco.alloisio@hunimed.eu (M.A.); 4 Department of Biomedical Science, Humanitas University, Rozzano, 20089 Milan, Italy; 5 Thoracic Surgery Unit, New Hospital of Legnano, ASST Ovest, 20025 Milan, Italy; michela.solinas@asst-ovestmi.it; 6 Faculty of Medicine and Surgery, Vita-Salute San Raffaele University, 20132 Milan, Italy

**Keywords:** robotic surgery, lung cancer, early stage, quality of life, postoperative pain

## Abstract

We compare the perioperative course, postoperative pain, and quality-of-life (QOL) in patients undergoing anatomic resections of early-stage lung cancer by means of robotic surgery (RATS), video-assisted thoracic surgery (VATS), or muscle-sparing thoracotomy (OPEN); 169 consecutive patients with known/suspected lung cancer, candidates to anatomic resection, were enrolled in a single-center prospective study from April 2016 to December 2018. EORTC QLQ-C30 and QLQ-LC13 scores were obtained preoperatively and, at three time points, postoperatively. RATS and VATS groups were matched for ASA scores, while RATS and open surgery were matched for gender, ASA score, cancer stage, and tumor size; 58 patients underwent open surgery, 58 had VATS, and 53 had RATS. Hospital stay was shorter after RATS than OPEN (median 4.5 versus 5; *p* = 0.047). Comparing matched RATS and VATS groups, the number of hilar lymph nodes and nodal stations removed was significantly higher in the former approach (*p* = 0.01 vs. *p* < 0.0001); conversely, pain at 2 weeks was slightly lower after VATS (*p* = 0.004). No significant difference was observed in conversions, complications, duration of surgery, and postoperative hospitalization. The robotic approach was superior to OPEN in terms of QOL, pain, and length of postoperative stay and showed improved lymph node dissection compared to VATS.

## 1. Introduction

Many pieces of evidence from retrospective and randomized controlled trials suggest that using minimally invasive approaches for the treatment of early-stage lung cancer is related to many clinical advantages in terms of perioperative outcomes with respect to open surgery [1,2,3,4,5,6,7]. In the randomized trial published by Bendixen in 2017 [8], video-assisted thoracic surgery (VATS) was shown to be superior in terms of pain and quality-of-life (QOL). However, manual videothoracoscopic surgery has several technical limitations, including long rigid instruments and a suboptimal 2D view, resulting in discomfort for the surgeons, a long-lasting learning curve, and, possibly, a suboptimal mediastinal lymphadenectomy extension [9].

Robotic surgery using the da Vinci system represents a technological evolution of the videothoracoscopic approach and is advantageous in terms of a better view of the operative field (3D instead of 2D), more intuitive use of the tools, and finer instrument precision, with a wider range of movements that is superior to that of the human hand [10,11].

These technical advantages have led to a great diffusion of the procedure among thoracic surgeons, especially those used to adopting open surgery for lobectomies. In the US, according to the registry of the Agency of Health Care Research and Quality (AHRQ), robotic lobectomies increased from 1% in 2010 to 18% in 2016; moreover, in the same time interval, a similar reduction in open cases (from 68% to 49%) was observed, while they reported stability in VATS procedures (31% versus 33%).

According to retrospective studies, in high-volume centers, robotic-assisted lobectomies seem to be associated with shorter hospitalization and reduced complication rates compared to muscle-sparing thoracotomy (OPEN), with lower conversion rates and 30-days morbidity compared to VATS [12]. However, given the relatively recent introduction of the robotic system in thoracic surgery, very little comparative data are available on long-term outcomes and quality of life [13].

In this prospective study, we compare the perioperative course, postoperative pain, and quality of life in lung cancer patients undergoing anatomic resection for early-stage NSCLC by robotic surgery (RATS), video-assisted thoracic surgery (VATS), or muscle-sparing thoracotomy (OPEN).

## 2. Materials and Methods

### 2.1. Study Design and Participants

From April 2016 to December 2018, we prospectively enrolled consecutive patients with suspected or diagnosed clinical stage I and II lung cancer who were candidates for radical surgical resection (ASA 1–3). Exclusion criteria were severe heart disease, renal impairment (creatinine >2.5), any other serious comorbidities according to the investigator, recent oncologic history (another malignant tumor in the previous 2 years), and previous chest surgery.

This study was approved by the Ethics Committee of our institute (No. 1524), and all patients signed an informed consent form. The study was also registered on Clinicaltrial.gov with the number NCT04353349. Preoperative staging included contrast-enhanced total body CT and FDG-PET. The standard functional evaluation included ECG, cardiological evaluation, pulmonary function tests, and anesthesiologic evaluation. The diffusion test for carbon monoxide has been systematically used since April 2017. Whenever required, additional tests were introduced, e.g., stress test, heart ultrasound, or pulmonary scintigraphy. Staging and functional exams were carried out within 8 weeks of surgery. In the case of suspicious mediastinal nodes, EBUS or mediastinoscopy was performed before resection. A preoperative diagnosis was obtained by CT-driven needle biopsy in the majority of patients. In the absence of a preoperative diagnosis, intraoperative lung cancer confirmation was obtained through frozen section.

### 2.2. Operative Approaches

All procedures were performed under general anesthesia, with patients in the lateral decubitus position. In most VATS lobectomies, a biportal approach was used (rarely uni- or triportal), with a 3-to-4 cm utility incision (without rib spreading) using a 30° 2D camera.

Conversely, a 4-port approach was used in the case of robotic lobectomies or segmentectomies, with a 3-cm utility incision without rib spreading and no routine CO_2_ insufflation. DaVinci Robotic System Xi was used with a 30° camera and standard endoscopic staplers [11,12,13,14,15].

The OPEN approach is a standard anterior muscle-sparing thoracotomy (12 to 15 cm).

The choice of approach mainly depended on robot availability and the surgeon’s preference (with one surgeon performing preferably robotic surgery, two surgeons performing VATS preferably, and one performing mainly OPEN).

Lymph node dissection was carried out according to International Association of the Study of Lung Cancer (IASLC) recommendations, i.e., with the removal of a minimum of 6 nodes and 3 mediastinal stations, including the subcarinal one [16]. One or two drainages were inserted, depending on the surgeon’s choice and the approach, with the OPEN surgeon using two chest tubes more frequently.

### 2.3. Perioperative Care

In patients undergoing VATS or robotic minimally invasive surgery, an intercostal block with chirocaine is performed from the third to the eighth intercostal space. In patients undergoing OPEN surgery, a peridural catheter is inserted unless specific contraindications prevent its use. All patients were administered postoperative paracetamol and ibuprofen at fixed times, adding an opiate in subjects with poor pain control. In the case of a prolonged air leak, a Heimlich valve is applied, and discharge is scheduled between the fifth and eighth postoperative day, providing there is an absence of clinical contraindications.

### 2.4. Assessment of Pain, Respiratory Function, and Quality of Life

Pain assessment was rated numerically using an 11-point scale (from 0 to 10), the ENV pain score, and the “Brief Pain Inventory” questionnaire. Quality of life was examined by means of two questionnaires: EORTC QLQ-C30 (version 3.0) [17] and EORTC QLQ-LC13 [18]. EORTC QLQ-C30 is a 30-item questionnaire assessing six functional domains of quality of life (physical function, emotional function, cognitive function, social function, and role function) and nine symptom domains (fatigue, pain, nausea and vomiting, dyspnoea, insomnia, appetite, constipation, diarrhea, and financial difficulties). EORTC-Quality of Life Questionnaire-Lung Cancer 13 (QLQ-LC13) was the first document developed in conjunction with the EORTC core Quality-of-Life (QOL) questionnaire.

The questionnaires were completed at 5 time points, namely, the day before surgery, Postoperative Day 3 (or at discharge, if earlier), and at 2 weeks, 6 months, and 12 months after surgery, during outpatient check-ups and follow-up visits.

### 2.5. Statistical Analysis

The sample size was calculated based on the primary endpoint, which was the prospective evaluation of the quality of life (QOL). Fifty-one patients operated on in each group provided 80% power to detect an 8-point difference in mean QLQ-C30 “pain scores” between any two study groups, at 2 weeks and 5% level of significance, assuming a coefficient of variability of 0.5 and using a two-sided two-sample *t*-test (the null hypothesis is that in both groups, the mean pain scores are 32, and the alternative hypothesis is that the mean pain score of the robotic group is 24, with estimated group standard deviations of 16 and 12).

Assuming that patients in the three intervention groups have similar QOL before intervention and a constant coefficient of variability of [cv = sd/mean] = 0.5 for each of the QOL scores, the sample size was determined by assuming differences in selected QOL domains 2 weeks after intervention between robotic, VATS-assisted and open resection groups, similar to that reported by Balduyck et al. after anterior mediastinal mass resection [19].

The Fisher exact test and the nonparametric Wilcoxon test were used to assess the differences in the distribution of categorical and continuous characteristics between the patient groups. For ordinal variables (tumor stage and tumor size), we used the Mantel–Haenszel test to assess significant trends across the groups. To reduce the imbalance in preoperative features (but not to decrease the sample size too much), we performed two different matched analyses: Robot versus VATS, matched for ASA score, and Robot versus OPEN, matched for gender, ASA score, stage, dimension >50 mm, and never-smoker status. All analyses were two-tailed, and *p*-values <0.05 were considered significant. Analyses were performed with the SAS software (version 9.4, Cary, NC, USA).

## 3. Results

From April 2016 to December 2018, 186 consecutive patients were enrolled in the study. Among these, 17 were excluded because of benign disease or withdrawal of consent, with a final number of 169 evaluated patients: 58 receiving OPEN, 58 VATS, and 53 RATS, respectively.

The prematched demographics and comorbidities of the three intervention groups are listed in Table 1. The three groups differed in ASA score, gender, stage, tumor dimension, and smoking status.

After the first round of matching, we compared 30 RATS and 30 OPEN cases. Baseline patient characteristics in the RATS and OPEN matched groups are reported in Table S1. Table 2 reports the intervention and tumor characteristics of the matched groups. Hospital stay was shorter after RATS than after OPEN surgery (median 4.5 versus 5; *p* = 0.047) while the median duration of surgery was longer for RATS than for OPEN (182 and 122 min, *p* = 0.0002), and the number of chest tubes was higher for OPEN than for RATS (*p* < 0.0001). Global health status and thoracic pain L13 were significantly improved after RATS than after the OPEN approach at 2 time points (at discharge (*p* = 0.03 and 0.0007) and at 12 months (*p* = 0.026 and 0.0025), respectively (Table 3)). No difference was observed in extension of lymph node dissection, radicality of surgery, and complications (Table 4).

**Figure 1:**
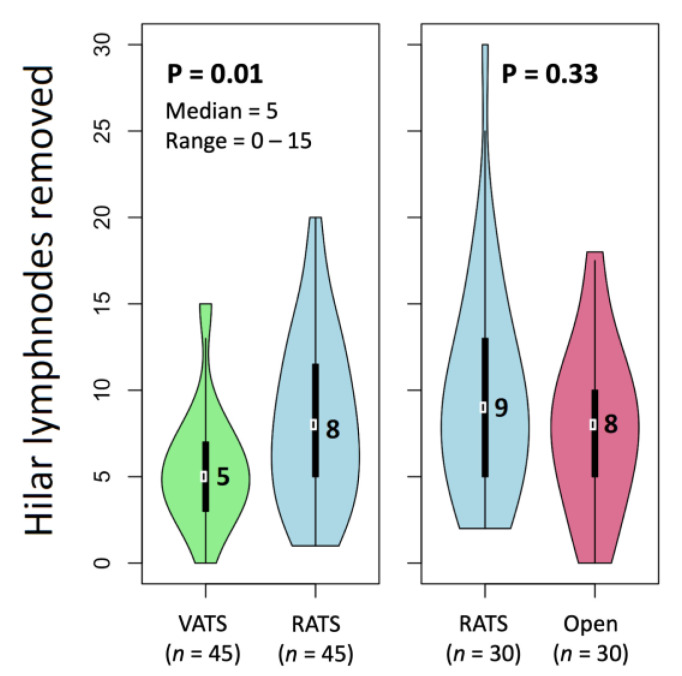
Differences between hilar lymph nodes resected in the different methods.

In the second matched analysis, we compared 45 VATS and 45 RATS cases. Baseline patient characteristics in the RATS and VATS matched groups are presented in Table S2, while intervention and tumor characteristics are reported in Table S3. When comparing these groups, a significantly higher median number of hilar lymph nodes (8 versus 5, *p* = 0.01) and stations (5 versus 4, *p* < 0.0001) was removed in the case of RATS (Table 5 and Figure 1). However, pain severity scores and pain interference scores at 2 weeks were lower for VATS than for RATS (*p* = 0.004; *p* = 0.01); the number of patients consuming pain killers did not differ between groups (Table 6). No significant difference was observed in conversions, complications, duration of surgery, and postoperative stay (Table 6).

## 4. Discussion

In this prospective study, we compare three different surgical approaches for the treatment of early-stage lung cancer in the same institute, focusing our analysis on postoperative pain and quality of life. We therefore separately matched patients who underwent RATS versus OPEN and RATS versus VATS.

Considering the first matched group, our analysis showed a lower pain score (LC13) and improved quality of life (global health status and Lung Cancer Scale) in the RATS group at 2 time points: at hospital discharge and 12 months after surgery; this was observed even though invasive epidural analgesia was administered only to patients undergoing OPEN surgery. Furthermore, in the RATS group, hospitalization was significantly shorter, with a lower number of chest tubes inserted and a similar extension of lymph node dissection and complications. Still, duration of surgery within the RATS group was longer compared to the OPEN approach.

When comparing matched RATS versus VATS groups, we observed two main differences: an improved lymph node dissection in the RATS group and a better pain profile in the VATS approach.

The most relevant result of this study was the documented superiority of the RATS approach compared to VATS in terms of lymph node dissection extension: a significantly higher median number of removed hilar lymph nodes and lymph node stations was observed, suggesting the more advantageous profile of the robotic approach in terms of oncological radicality. Long-term survival was not assessed, and, at the moment, it is not possible to establish whether there is a real advantage in terms of survival or local recurrences for the robotic arm. A longer follow-up is required to observe these aspects. It is known that the main benefit of robotic surgery is the simpler and more intuitive execution of lymphadenectomy at both hilar and mediastinal stations [14,20]. A recent paper by Kneuertz et al. showed that robotic lobectomy for clinical N0/N1 NSCLC was associated with similar lymph node upstaging to the OPEN approach but increased upstaging compared to VATS [21], confirming the results of Wilson et al., published in 2014 [22]. The main limitation of a previous randomized study on VATS versus OPEN lobectomy by Bendixen and colleagues was that they did not present any result on lymph node dissection extension, only focusing on the main goal of the study, which was the evaluation of quality of life and postoperative pain [8].

Concerning postoperative pain, the VATS approach showed a more favorable pain control profile at 2 weeks (the primary endpoint). Although significant, the difference in pain was very small, and its clinical relevance is not clear. The following variables were different: pain severity score, pain interference score, thoracic pain LC13, and thoracic pain LC13 (rescaled 0–100). At 12 months, only one of these scores was superior in VATS, with an average value of pain lower than 1 in both groups. This result was expected, as two additional trocars are used in RATS with respect to VATS. However, it is our personal opinion that the increased pain in RATS is not exclusively related to the higher number of ports used but to the position of the utility incision: in VATS, in fact, the utility incision is 2 to 3 cm more lateral than in RATS, and this position spares the skin’s sensitive nerves. Those patients experiencing postoperative discomfort have indeed complained of abdominal wall paresthesia, localized on the right hypochondrium, beneath the 12th rib. In addition, VATS procedures are mainly performed using a biportal trocarless approach with two skin retractors.

To manage this side effect of RATS, in the future, we may improve the volume and composition of local anesthetic infiltration and, possibly, place the utility incision slightly downwards and laterally [23]. The use of a subxiphoid incision for the removal of the specimen and the robotic arm instead of the fourth intercostal space or the use of a subdiaphragmatic incision similar to the Dylewski technique may also be a potential solution to minimizing postoperative pain [24]. To this end, comparative studies on different variants of robotic approaches should be implemented in the new ESTS robotic registry, in which the results of different RATS approaches will be reported. Notably, despite the increased pain in the RATS group, the number of subjects taking analgesics was identical at all time points in both VATS and RATS groups.

Another observation coming from the analysis of the real-world prospective enrollment is the difference in preoperative variables between RATS and VATS as far as the ASA score is concerned: being worse in the robotic group, it reflects the wider indications of RATS compared to VATS in the selection of candidates for minimally invasive procedures. ASA scores in RATS were similar to that of OPEN surgery, again highlighting the wider pool of eligible patients with respect to VATS, in which the selection tends to be limited to fitter patients. A recent paper comparing RATS and open surgery in a cohort of 599 patients, including 189 subjects at high risk for limited pulmonary function, showed a lower pulmonary complication rate after RATS versus the OPEN approach (22 versus 32%) [25]; this difference in complication rate was even more remarkable in the high-risk group (28% vs. 45%, *p* = 0.02).

The strength of our work is that it is the first prospective study assessing pain and QOL at different time points in three different techniques in the same center, with adequate power calculation. In addition, all patients were staged in a similar way, and all received preoperative PET scans, brain CTs, and similar postoperative management of complications, chest tubes, urine catheters, and periodic follow-ups. This is the representation of real-world clinical activity in a prospective observation study.

Limitations included the inhomogeneous initial distribution of preoperative risk factors in the three surgical groups due to the absence of randomization. These differences were mitigated by the selection criteria adopted prospectively and the matching between groups performed before the statistical analysis. In addition, the dosage of drugs and additional painkillers was not recorded. It is probable that the patients of the OPEN approach were treated with additional drugs other than epidural catheters, but the dosage was not recorded. Similarly, we were not able to record the side effects of opioid treatment as the sedation score was assessed with the Ramsay scale [25] and there are other complications related to epidural analgesia.

Some studies have compared postoperative pain in uniportal VATS versus triportal VATS; up to now, there has been no evidence of a patient advantage regarding perioperative pain control for uniportal VATS [26,27]. Similarly, the paper by Yang et al. [28], comparing uniportal VATS versus RATS, showed similar analgesic usage between the two approaches, with RATS being superior in controlling bleeding and achieving complete lymphadenectomy.

## 5. Conclusions

The robotic approach is superior in terms of lymph node dissection when compared to VATS lobectomy in the real world, based on this prospective study, even though the implication in terms of local recurrence or oncological outcome needs to be established with a longer follow-up.

## Figures and Tables

**Table 1:** Baseline patient characteristics in the three intervention groups before matching.

	VATS	RATS	OPEN	VATS vs. RATS	VATS vs. OPEN	RATS vs. OPEN
	N (%)	N (%)	N (%)	*p*-Value	*p*-Value	*p*-Value
**Total**	**58 (100)**	**53 (100)**	**58 (100)**			
**Gender**						
Male	31 (53.5)	28 (52.8)	36 (62.1)			
Female	27 (46.5)	25 (47.2)	22 (37.9)	1.00	0.45	0.34
**Age (years)**						
Median [range]	70 [43–85]	69 [43–81]	69 [30–81]	0.73	0.89	0.66
<50	2 (3.5)	3 (5.7)	2 (3.5)			
50–59	10 (17.2)	7 (13.2)	8 (13.8)			
60–69	16 (27.6)	19 (35.8)	19 (32.8)			
70–79	28 (48.3)	22 (41.5)	25 (43.1)			
80+	2 (3.4)	2 (3.8)	4 (6.9)	0.84	0.87	0.93
**BMI (kg/m^2^)**						
Median [range]	25.1 [17.0–39.2]	25.4 [19.8–52.3]	24.7 [17.5–38.7]	0.68	0.23	0.47
**FEV1%**						
Median [range]	95 [50–143]	95 [61–139]	84 [28–154]	0.55	**0.05**	**0.02**
<80% predicted	12 (21.0)	12 (22.6)	21 (36.2)			
≥80% predicted	45 (79.0)	41 (77.4)	37 (63.8)	1.00	0.10	0.15
**FEV1/FVC**						
Median [range]	0.75 [0.58–1.30]	0.77 [0.63–1.35]	0.74 [0.39–1.03]	0.11	0.24	**0.02**
DLCO% *						
Median [range]	81 [45–109]	74 [40–119]	73 [49–108]	0.47	0.11	0.49
<80% predicted	13 (46.4)	15 (62.5)	15 (75.0)			
≥80% predicted	15 (53.6)	9 (37.5)	5 (25.0)	0.28	0.08	0.52
**ASA score**						
1	4 (7.0)	1 (1.9)	1 (1.8)			
2	49 (86.0)	40 (75.5)	50 (89.3)			
3	4 (7.0)	12 (22.6)	5 (8.9)	**0.03**	0.47	0.10
**Cardiological evaluation**						
Negative	49 (87.5)	41 (80.4)	48 (84.2)			
Positive	7 (12.5)	10 (19.6)	9 (15.8)	0.43	0.79	0.62
						
**Pack-years**						
Never	19 (32.8)	14 (26.4)	6 (10.3)			
<30 pack-years	10 (17.2)	13 (24.5)	13 (22.4)			
≥30 pack-years	26 (44.8)	22 (41.5)	35 (60.3)			
Unknown	3 (5.2)	4 (7.6)	4 (6.9)	0.71	**0.03**	0.11

* Missing for few patients.

**Table 2:** Intervention and tumor characteristics in the matched robotic surgery (RATS) and muscle-sparing thoracotomy (OPEN) intervention groups.

	RATS	Open	RATS vs. Open
	N (%)	N (%)	*p*-Value
**Total**			
**Anesthesia**			
Intercostal	27	1	
Morphine	0	1	
Peridural	1	26	**<0.00001**
**Conversion**			
No	27	n/a	
Yes	3	n/a	n/a
**Side**			
Right	17	11	
Left	13	19	0.20
**Lobe**			
Inferior	11	10	
Medium	2	2	
Superior	17	18	1.00
**Pleural adherence**			
Absent	14	19	
Light	2	3	
Moderate	5	4	
Strong	3	3	0.94
**Fissure**			
Absent	2	0	
Partial	17	24	
Complete	5	3	0.23
**Extent of surgery**			
R0	30	29	
R1	0	1	0.24
**LN dissection**			
No	0	1	
Sampling	4	7	
Radical	26	22	0.33
			
**Duration of surgery**			
Median [range]	182 [82–278]	122 [78–223]	**0.0002**
<120 min	4	14	
120–149 min	6	9	
150–179 min	5	6	
≥180 min	15	1	**0.0001**
**Blood loss**			
No	28	28	
Yes	2	2	1.00
**N** **^o^** **thoracic drains (DT)**			
1	26	9	
2	4	21	**<0.0001**
**HISTOPATHOLOGY**			
**Tumor size (cm)**			
Median [range]	21 [8–73]	26 [4–55]	0.21
0–19 mm	11	9	
20–29 mm	10	9	
30–49 mm	6	9	
≥50 mm	3	3	0.87
**Tumor stage (TNM 8th)**			
I	19	19	
II	7	7	
III	4	4	
IV	0	0	Matching
**Histology**			
ADK	23	22	
SCC	5	5	
other	2	3	1.00
**Tumor grade**			
G1	2	0	
G2	17	16	
G3	6	9	0.37

**Table 3:** QLQ-C30/LC13 and brief inventory pain in the matched RATS and OPEN intervention groups.

	RATS	Open	RATS vs. Open
			*p*-Value
**Preop**			
Global health status	83.3 [16.7–100]	93.3 [16.7–100]	0.34
Functional score	91.1 [77.8–100]	91.1 [37.8–100]	0.76
Symptom scale	6.4 [0.0–23.1]	2.6 [0.0–43.6]	**0.32**
Lung Cancer Scale	5.6 [0.0–16.7]	8.3 [0.0–30.4]	0.21
Takes medicine for pain	3 (10.3%)	7 (23.3%)	0.30
Pain severity score	0.0 [0.0–4.0]	0.0 [0.0–4.0]	0.54
Pain interference score	0.0 [0.0–4.0]	0.0 [0.0–6.5]	0.31
Thoracic pain LC13	1.00 ± 0.00	1.17 ± 0.38	**0.02**
Thoracic pain LC13 (rescaled 0–100)	25.0 ± 0.0	29.3 ± 9.5	**0.02**
**Hospital discharge**			
Global health status	75 [33–100]	66.7 [8.3–100]	**0.03**
Functional score	86.7 [40.5–98.4]	84.4 [31.1–95.6]	0.23
Symptom scale	15.4 [0.0–53.9]	25.6 [0.0–59.0]	0.14
Lung Cancer Scale	11.1 [2.8–30.3]	15.9 [0.0–55.6]	**0.036**
Takes medicine for pain	23 (92.0%)	24 (84.2%)	0.43
Pain severity score	2.0 [0.0–7.7]	3.3 [0.3–7.3]	0.28
Pain interference score	3.3 [0.3–8.8]	3.4 [1.0–7.4]	0.66
Thoracic pain LC13	1.20 ± 0.85	1.90 ± 1.06	**0.007**
Thoracic pain LC13 (rescaled 0–100)	30.0 ± 21.2	47.5 ± 26.5	**0.007**
**2-week visit**			
Global health status	66.7 [16.7–100]	54.2 [16.7–91.7]	0.13
Functional score	82.2 [42.2–100]	82.8 [48.9–100]	0.47
Symptom scale	15.4 [0.0–46.2]	20.5 [0.0–53.9]	0.07
Lung Cancer Scale	11.1 [2.8–38.9]	13.9 [2.8–55.6]	0.15
Takes medicine for pain	20 (71.4%)	23 (82.1%)	0.53
Pain severity score	2.0 [0.0–7.7]	2.0 [0.0–5.0]	0.97
Pain interference score	1.1 [0.0–9.0]	1.7 [0.0–6.4]	0.82
Thoracic pain LC13	1.47 ± 0.63	1.77 ± 0.94	0.19
Thoracic pain LC13 (rescaled 0–100)	36.7 ± 15.8	44.3 ± 23.5	0.19
**6-month visit**			
Global health status	83.3 [33.3–100]	75.0 [16.7–100]	0.30
Functional score	90.0 [57.1–100]	86.7 [20.0–97.8]	0.34
Symptom scale	11.5 [0.0–48.7]	7.7 [0.0–64.1]	0.65
Lung Cancer Scale	9.7 [0.0–22.2]	11.1 [2.8–27.3]	0.13
Takes medicine for pain	4 (20.0%)	5 (23.8%)	1.00
Pain severity score	0.0 [0.0–4.0]	1.0 [0.0–4.0]	0.08
Pain interference score	0.1 [0.0–8.8]	0.0 [0.0–8.0]	0.58
Thoracic pain LC13	0.93 ± 0.69	1.00 ± 0.91	0.98
Thoracic pain LC13 (rescaled 0–100)	23.3 ± 17.3	25.0 ± 22.8	0.98
**12-month visit**			
Global health status	83.3 [33.3–100]	62.5 [8.3–100]	**0.026**
Functional score	88.9 [51.1–100]	77.8 [31.1–100]	0.12
Symptom scale	7.7 [0.0–28.2]	12.8 [0.0–59.0]	0.12
Lung Cancer Scale	8.3 [0.0–36.0]	13.9 [0.0–47.2]	**0.03**
Takes medicine for pain	3 (18.8%)	8 (31.8%)	0.28
Pain severity score	0.2 [0.0–5.0]	1.7 [0.0–5.7]	0.17
Pain interference score	0.1 [0.0–6.5]	1.4 [0.0–8.0]	0.06
Thoracic pain LC13	0.63 ± 0.72	1.13 ± 0.90	**0.025**
Thoracic pain LC13 (rescaled 0–100)	15.8 ± 18.0	28.3 ± 22.5	**0.025**

**Table 4:** Perioperative outcome and pathological results in the matched RATS and OPEN intervention groups.

	RATS	Open	RATS vs. Open
	N (%)	N (%)	*p*-Value
**Total**			
**Hilar LN removed**			
Median [range]	9 [2–30]	8 [0–18]	0.33
0–5	8	10	
6–10	11	13	
>10	10	7	0.68
**Positive hilar LN**			
0	25	25	
1+	5	5	1.00
**Med LN removed**			
Median [range]	6 [1–18]	5 [0–24]	0.44
0–5	14	16	
6–10	11	10	
>10	5	4	0.88
**Positive Med LN**			
0	26	27	
1+	3	3	1.00
**Stations removed**			
Median [range]	5 [3–6]	4 [2–8]	0.42
1–3	5	8	
4	9	10	
5–8	16	12	0.50
**ICU**			
No	28	24	
Yes	0	2	0.23
**Complication**			
No	18	19	
Yes	12	11	1.00
**Complications grade (max)**			
No	18	19	
G1–G2	9	5	
G3–G4	3	5	0.48
**Re-intervention**			
No	30	28	
Yes	0	2	0.49
**Hospital stay (postintervention)**			
Median [range]	4.5 [2–18]	5.0 [2–28]	0.08
2–3 days	11	3	
4–6 days	11	18	
7–13 days	6	4	

**Table 5:** Perioperative outcomes and pathological results in the matched RATS and VATS intervention groups.

	VATS	RATS	VATS vs. RATS
	N (%)	N (%)	*p*-Value
**Total**	**45 (100)**	**45 (100)**	
**Hilar LN removed**			
Median [range]	5 [0–15]	8 [1–20]	**0.01**
0–5	23 (57.5)	15 (34.9)	
6–10	14 (35.0)	15 (34.9)	
>10	3 (7.5)	13 (30.2)	**0.02**
**Positive hilar LN**			
0	39 (90.7)	39 (86.7)	
1+	4 (9.3)	6 (13.3)	0.74
**Med LN removed**			
Median [range]	5 [0–17]	6 [1–18]	0.06
0–5	24 (60.0)	20 (44.4)	
0–5	24 (60.0)	20 (44.4)	
6–10	12 (30.0)	18 (40.0)	
>10	4 (10.0)	7 (15.6)	0.35
Positive Med LN			
0	39 (90.7)	41 (93.2)	
1+	4 (9.3)	3 (6.8)	0.71
Stations removed			
Median [range]	4 [1–7]	5 [2–7]	0.001
1–3	13 (28.9)	7 (15.6)	
4	18 (40.0)	10 (22.2)	
5–8	14 (31.1)	28 (62.2)	0.01
ICU			
No	38 (95.0)	42 (97.7)	
Yes	2 (5.0)	1 (2.3)	0.61
Complication			
No	31 (68.9)	29 (64.4)	
Yes	14 (31.1)	16 (35.6)	0.82
Complications grade (max)			
No	31 (72.1)	29 (64.4)	
G1–G2	9 (20.9)	12 (26.7)	
G3–G4	3 (7.0)	4 (8.9)	0.74
Reintervention			
No	42 (95.5)	45 (100)	
Yes	2 (4.5)	0 (0.0)	0.24
Hospital stay (postintervention)			
Median [range]	4 [2–26]	4 [2–18]	0.85
2–3 days	12 (26.7)	17 (37.8)	
4–6 days	26 (57.8)	16 (35.6)	
7–13 days	6 (13.3)	10 (22.2)	
≥14 days	1 (2.2)	2 (4.4)	0.19

**Table 6:** QLQ-C30/LC13 and brief inventory pain in the matched RATS and VATS interventional groups.

	VATS	RATS	VATS vs. RATS
			*p*-Value
**Preop**	N = 45	N = 45	
Global health status	83 [33–100]	83 [0–100]	0.48
Functional score	93 [58–100]	91 [27–100]	0.23
Symptom scale	2.6 [0.0–27.8]	5.1 [0.0–56.4]	**0.03**
Lung Cancer Scale	3.0 [0.0–27.8]	5.6 [0.0–41.7]	0.24
Takes medicine for pain	7 (16.7)	6 (13.6)	0.77
Pain severity score	0.0 [0.0–3.7]	0.0 [0.0–8.0]	0.65
Pain interference score	0.0 [0.0–3.5]	0.0 [0.0–7.0]	0.56
Thoracic pain LC13	1.16 ± 0.52	1.09 ± 0.36	0.47
Thoracic pain LC13 (rescaled 0–100)	29.0 ± 13.0	27.2 ± 9.0	0.47
**Hospital discharge**	N = 42	N = 42	
Global health status	54 [0–100]	67 [25–100]	0.13
Functional score	83 [38–100]	82 [27–98]	0.76
Symptom scale	17 [0–67]	24 [0–69]	0.36
Lung Cancer Scale	11 [0–47]	13 [3–39]	0.64
Takes medicine for pain	35 (87.5)	36 (92.3)	0.71
Pain severity score	2.7 [0.0–8.0]	2.5 [0.0–7.7]	0.51
Pain interference score	2.5 [0.0–8.1]	3.4 [0.3–8.8]	0.38
Thoracic pain LC13	1.38 ± 0.75	1.36 ± 0.86	0.69
Thoracic pain LC13 (rescaled 0–100)	34.5 ± 18.8	34.0 ± 21.5	0.69
**2-week visit**	N = 36	N = 42	
Global health status	67 [0–100]	67 [0–100]	0.46
Functional score	89 [58–100]	80 [7–100]	**0.03**
Symptom scale	15 [0–36]	21 [0–75]	0.09
Lung Cancer Scale	11 [3–36]	12 [3–52]	0.47
Takes medicine for pain	24 (70.6)	30 (73.2)	1.00
Pain severity score	1.3 [0.0–5.3]	2.7 [0.0–9.0]	**0.004**
Pain interference score	0.8 [0.0–6.3]	3.1 [0.3–9.0]	**0.01**
Thoracic pain LC13	1.18 ± 0.78	1.64 ± 0.88	**0.02**
Thoracic pain LC13 (rescaled 0–100)	29.5 ± 19.5	41.0 ± 22.0	**0.02**
**6-month visit**	N = 34	N = 36	
Global health status	83 [17–100]	83 [0–100]	0.38
Functional score	90 [56–100]	87 [42–100]	0.15
Symptom scale	7 [0–31]	14 [0–51]	0.049
Lung Cancer Scale	6 [0–22]	11 [0–31]	0.28
Takes medicine for pain	6 (18.2)	6 (18.8)	1.00
Pain severity score	0.0 [0.0–6.0]	0.7 [0.0–6.0]	0.19
Pain interference score	0.0 [0.0–6.4]	0.3 [0.0–8.8]	0.06
Thoracic pain LC13	0.82 ± 0.58	1.11 ± 0.75	0.06
Thoracic pain LC13 (rescaled 0–100)	20.5 ± 14.5	27.8 ± 18.8	0.06
**12-month visit**	N = 29	N = 27	
Global health status	83 [0–100]	83 [33–100]	0.87
Functional score	93 [5–100]	87 [51–100]	0.46
Symptom scale	8 [0–28]	11 [0–36]	0.19
Lung Cancer Scale	8 [0–31]	8 [0–31]	0.96
Takes medicine for pain	10 (35.7)	7 (25.9)	0.56
Pain severity score	0.0 [0.0–3.0]	0.7 [0.0–6.0]	**0.02**
Pain interference score	0.0 [0.0–5.0]	0.3 [0.0–6.9]	0.15
Thoracic pain LC13	0.69 ± 0.60	0.80 ± 0.81	0.74
Thoracic pain LC13 (rescaled 0–100)	17.3 ± 15.0	20.0 ± 20.3	0.74

## Data Availability

The data presented in this study are available on request from the corresponding author. The data are not publicly available.

## Supplementary Materials

The following are available online at https://www.mdpi.com/article/10.3390/jcm10081687/s1, Table S1: Baseline patients’ characteristics in the RATS and OPEN groups matched for gender, ASA score, stage, dimension and smoking., Table S2: Baseline patients’ characteristics in the matched VATS and RATS intervention groups, Table S3: Intervention and tumor characteristics in matched VATS and RATS intervention groups.
