# Supplementary material for: Quality of Life, Postoperative Pain, and Lymph Node Dissection in a Robotic Approach Compared to VATS and OPEN for Early Stage Lung Cancer
Source: J Clin Med. 2021 Apr 14;10(8):1687. doi: 10.3390/jcm10081687 (PMC8071041; doi:10.3390/jcm10081687)
Supplement: Supplementary file 1 [file jcm-10-01687-s001.pdf]

Supplementary table 1. Baseline patients' characteristics in the RATS and OPEN groups matched for gender, ASA score, stage, dimension and smoking.

		RATS<br>N (%)	Open<br>N (%)	RATS vs Open<br>P-value
**Total**		**30 (100)**	**30 (100)**	
**Gender**				
	Male	20 (66.7)	20 (66.7)	
	Female	10 (33.3)	10 (33.3)	Matching
**Age (years)**				
	Median [range]	69 [48-80]	67 [54-79]	0.81
	<50	1 (3.3)	0 (0.0)	
	50-59	4 (13.3)	6 (20.0)	
	60-69	12 (40.0)	11 (36.7)	
	70-79	12 (40.0)	13 (43.3)	
	80+	1 (3.3)	0 (0.0)	0.86
**BMI (kg/m^2)**				
	Median [range]	25.8 [19.8-52.3]	24.4 [19.2-38.7]	0.08
	Normal weight	11 (36.7)	18 (60.0)	
	Overweight	12 (40.0)	11 (36.7)	
	Obese	7 (23.3)	1 (3.3)	**0.05**
**FEV1%**				
	Median [range]	100 [63-139]	90 [62-124]	0.07
	<80% predicted	7 (23.3)	11 (36.7)	
	≥80% predicted	23 (76.7)	19 (63.3)	0.40
**FVC%**				
	Median [range]	101 [64-129]	97 [71-134]	0.50
	<80% predicted	3 (10.0)	3 (10.0)	
	≥80% predicted	27 (90.0)	27 (90.0)	1.00
**FEV1/FVC**				
	Median [range]	0.77 [0.63-1.03]	0.74 [0.39-1.03]	0.22
	<70%	3 (10.0)	8 (26.7)	
	≥70%	27 (90.0)	22 (73.3)	0.18
**DLCO%****				
	Median [range]	75 [66-92]	66 [49-82]	0.09
	<80% predicted	6 (66.7)	7 (87.5)	
	≥80% predicted	3 (33.3)	1 (12.5)	0.57
**ASA score**				
	1	1 (3.3)	1 (3.3)	
	2	28 (93.3)	28 (93.3)	
	3	1 (3.3)	1 (3.3)	matching
**Cardiological evaluation**				
	Negative	23 (82.1)	25 (83.3)	
	Positive	5 (17.9)	5 (16.7)	1.00
**Smoking**				
	Never	2 (6.7)	2 (6.7)	
	Former	18 (60.0)	11 (36.7)	
	Current	10 (33.3)	17 (56.7)	0.21
**Pack-years**				
	Never	2 (6.7)	2 (6.7)	
	<30 pack-years	10 (33.3)	8 (26.7)	
	≥30 pack-years	14 (46.7)	18 (60.0)	
	Unknown	4 (13.3)	2 (6.7)	0.76

** Missing for few patients

Supplementary table 2. Baseline patients' characteristics in the matched VATS and RATS intervention groups

		**VATS** **N (%)**	**RATS** **N (%)**	**VATS vs RATS P-value**
**Total**		**45 (100)**	**45 (100)**	
**Gender**				
	Male	27 (60.0)	25 (55.6)	
	Female	18 (40.0)	20 (44.4)	0.83
**Age (years)**				
	Median [range]	70 [43-85]	68 [43-80]	0.43
	<50	2 (4.4)	3 (6.7)	
	50-59	7 (15.6)	6 (13.3)	
	60-69	12 (26.7)	18 (40.0)	
	70-79	22 (48.9)	17 (37.8)	
	80+	2 (4.4)	1 (2.2)	0.68
**BMI (kg/m^2)**				
	Median [range]	25 [17-39]	25 [20-32]	0.34
	Normal weight	22 (48.9)	20 (44.4)	
	Overweight	13 (28.9)	19 (42.2)	
	Obese	10 (22.2)	6 (13.3)	0.33
**FEV1%**				
	Median [range]	95 [40-143]	99 [61-139]	0.23
	<80% predicted	10 (22.7)	9 (20.0)	
	≥80% predicted	34 (77.3)	36 (80.0)	0.80
**FVC%**				
	Median [range]	99 [64-139]	99 [62-129]	0.71
	<80% predicted	8 (18.2)	4 (8.9)	
	≥80% predicted	36 (81.8)	41 (91.1)	0.23
**FEV1/FVC**				
	Median [range]	0.75 [0.58-1.30]	0.77 [0.63-1.03]	0.08
	<70%	12 (27.3)	5 (11.1)	
	≥70%	32 (72.7)	40 (88.9)	0.06
**DLCO%****				
	Median [range]	81 [45-109]	74 [40-92]	0.32
	<80% predicted	10 (43.5)	11 (68.7)	
	≥80% predicted	13 (56.5)	5 (31.3)	0.19
**RISK****				
	Low (FEV1P≥80 and DLCOp≥80)	12 (52.2)	5 (31.2)	
	Intermediate (FEV1P<80 or DLCOp<80)	9 (39.1)	10 (62.5)	
	High (FEV1P<60 or DLCOp<60)	2 (8.7)	1 (6.3)	0.34
**ASA score**				
	1	1 (2.2)	1 (2.2)	
	2	40 (88.9)	40 (88.9)	
	3	4 (8.9)	4 (8.9)	matching
**Cardiological evaluation**				
	Negative	37 (86.0)	38 (86.4)	
	Positive	6 (14.0)	6 (13.6)	1.00
**Smoking**				
	Never	15 (33.3)	13 (28.9)	
	Former	15 (33.3)	20 (44.4)	
	Current	15 (33.3)	12 (26.7)	0.60
**Pack-years**				
	Never	15 (33.3)	12 (26.7)	
	<30 pack-years	8 (17.8)	12 (26.7)	
	≥30 pack-years	19 (42.2)	17 (37.8)	
	Unknown	3 (6 .7)	4 (8.9)	0.76

** Missing for few patients

Supplementary table 3. Intervention and tumor characteristics in matched VATS and RATS intervention groups

		VATS N (%)	RATS N (%)	VATS vs RATS P-value
**Total**		**45 (100)**	**45 (100)**	
**Anesthesia**				
	Intercostal	38 (97.4)	42 (100)	
	Morphine	0 (0.0)	0 (0.0)	
	Peridural	1 (2.6)	0 (0.0)	0.48
**Conversion**				
	No	42 (93.3)	41 (91.1)	
	Yes	3 (6.7)	4 (8 .9)	1.00
**Side**				
	Right	29 (64.4)	28 (62.2)	
	Left	16 (35.6)	17 (37.8)	1.00
**Lobe**				
	Inferior	19 (44.2)	15 (33.3)	
	Medium	3 (7.0)	6 (13.3)	
	Superior	21 (48.8)	24 (53.3)	0.45
**Pleural adherence**				
	Absent	24 (58.5)	23 (62.2)	
	Light	12 (29.3)	4 (10.8)	
	Moderate	5 (12.2)	6 (16.2)	
	Strong	0 (0.0)	4 (10.8)	**0.045**
**Fissure**				
	Absent	2 (4.9)	3 (8.1)	
	Partial	27 (65.9)	23 (62.2)	
	Complete	12 (29.3)	11 (29.7)	0.93
**Extent of surgery**				
	R0	36 (97.3)	41 (93.2)	
	R1	1 (2.7)	3 (6.8)	0.62
**LN dissection**				
	No	4 (8.9)	0 (0.0)	
	Sampling	4 (8.9)	5 (11.1)	
	Radical	37 (82.2)	40 (88.9)	0.17
	Other	-	-	
**Duration of intervention**				
	Median [range]	171 [83-334]	162 [82-278]	0.32
	<120 min	3 (6.8)	7 (15.6)	
	120-149 min	11 (25.0)	12 (26.7)	
	150-179 min	12 (27.3)	10 (22.2)	
	≥180 min	18 (40.9)	16 (35.6)	0.62
**Blood loss**				
	No	45 (100)	42 (93.3)	
	Yes	0 (0.0)	3 (6.7)	0.24
**N◦ thoracic drains (DT)**				
	1	32 (71.1)	40 (88.9)	
	2	13 (28.9)	5 (11.1)	0.06
**HISTOPATHOLOGY**				
**Tumor size (mm)**				
	Median [range]	2.2 [0.9-5.3]	2.1 [0.8-7.3]	0.66
	0-19 mm	16 (35.6)	17 (37.8)	
	20-29 mm	13 (28.9)	14 (31.1)	
	30-49 mm	14 (31.1)	10 (22.2)	
	≥50 mm	2 (4.4)	4 (8.9)	0.74
**Tumor stage (TNM 8th)**				
	I	31 (68.9)	31 (68.9)	
	II	9 (20.0)	9 (20.0)	
	III	5 (11.1)	4 (8.9)	
	IV	0 (0.0)	1 (2.2)	1.00
**Histology**				
	ADK	33 (73.3)	34 (75.6)	
	SCC	7 (15.6)	7 (15.6)	
	TC	5 (11.1)	4 (8.9)	1.00
	SAR	-	-	
**Tumor grade**				
	G1	5 (13.2)	4 (10.3)	
	G2	29 (76.3)	26 (66.7)	
	G3	4 (10.5)	9 (23.1)	0.36
